# Linguistically‐appropriate tools to assess cognition in Mandarin speakers

**DOI:** 10.1002/alz.088311

**Published:** 2025-01-03

**Authors:** William T. Hu, Michelle H. Chen, Mei‐Ling Li, Guibin J Su, Feng Vankee Lin

**Affiliations:** ^1^ Rutgers‐Robert Wood Johnson Medical School, New Brunswick, NJ USA; ^2^ Rutgers Institute for Health, Health Care Policy, and Aging Research, New Brunswick, NJ USA; ^3^ Stanford University, Stanford, CA USA

## Abstract

**Background:**

Assessment of cognition in older Chinese Americans currently relies on content‐translated instruments with limited considerations for logographic (vs. alphabetic) nature of Chinese, cultural experience (pre‐ and post‐immigration), speech rate, and multilingualism. This results in confusion between fluency tasks, systematic error in digit span, disparate familiarity with stimuli according to country of origin (e.g., Taiwan vs. China), and low overall ecological validity. Here we developed and tested new instruments on memory (logical memory & word list learning), executive function (culturally‐appropriate Trail Making Test B), and language (fluency based on character, phoneme/pinyin, and homonym) to account for these factors.

**Method:**

Bilingual (Mandarin and English, n = 50) and Mandarin‐only (n = 50) speakers were recruited in the NYC/New Jersey (Rutgers) and SF Bay (Stanford) areas. Bilingual adults completed both English and Mandarin cognitive testing for correlation, while monolingual speakers completed only Mandarin testing for reliability. Instruments in simplified and traditional Chinese were available for all, and each participant additionally underwent Clinical Dementia Rating, language proficiency(Mandarin, English), and brain MRI analysis.

**Result:**

Participants had a median age of 66 yr (range 56‐84), and monolingual participants had been in the US for less than bilingual participants (33 vs. 42 yr, p = 0.004). New Mandarin tests had very high reliability (median Cronbach’s alpha of 0.935 range 0.807‐0.969). Among bilingual participants, performance in English and Mandarin for language‐independent tests (Trail Making Test A, written Symbol Digit Substitution Test) and some language‐dependent (e.g., animal fluency, Trail Making Test B) tests had acceptable to high reliability (standardized Cronbach’s alpha 0.638‐0.838). Total learning and delayed recall of corpus‐adjusted (based on word use frequency in American English and Beijing Chinese) also had high reliability (0.681 and 0.774) between languages, while character‐guided and animal fluency in Mandarin (0.560, p = 0.004) had similar correlation as English‐based letter‐guided and animal fluency (0.600, p = 0.005). Logical memory in English was associated with English proficiency and learning of a list of English words, but not logical memory in Mandarin.

**Conclusion:**

Most novel Mandarin cognitive assessments showed high inter‐language reliability in bilingual older adults, and their diagnostic validity is being prospectively tested.

